# Comments on: Nuclear management in manual small incision cataract surgery by snare technique

**DOI:** 10.4103/0301-4738.64140

**Published:** 2010

**Authors:** Parthasarathi Roy

**Affiliations:** Haldia Sub-Divisional Government Hospital, Purba Medinipur, West Bengal- 721 602, India

Dear Editor,

I read with interest the article entitled "Nuclear management in manual small incision cataract surgery by snare technique" by Bhattacharya.[1] In the beginning of the article itself, it is stated "Keener in 1983 was the first to snare the nucleus into two halves and bring the fragments out through a sclerocorneal flap valve incision.[1]" On further search[2] I found that Gerald Keener in 1983 prepared a snare from an 18-19-G blunt-tipped needle and a 32-G stainless steel wire. After the nuclear prolapse into the anterior chamber, he positioned the wire loop around the nucleus. The loop was then shortened by pulling it back, resulting the nucleus to be divided into two. This is exactly the same technique described by Dr. Bhattacharya in his article. Therefore Dr. Bhattacharya is reviving a technique originally developed by Keener in 1983,[2] after a prolonged period of dormancy.

Dr. Bhattacharya has admitted in his article abstract that much evidence of this technique is not available in literature, as its popularity grew through live surgical workshops and small interactive conferences. However, can a video clip in a live surgical workshop predict postoperative endothelial count or intraocular pressure (IOP) three months postoperatively? Certainly not. Therefore I seriously doubt whether a new surgical technique which is meant to be applied on human beings should be accepted without a number of clinical (i.e. surgical) trials maintaining a proper research methodology. In an age-and sex-matched adequately sized sample, selected by predetermined inclusion and exclusion criteria, with standardized surgical technique, results of a number of intraoperative and postoperative parameters are to be analyzed and compared with other techniques, before being presented to peers for acceptance. The author did not do that. Let me put forward some related surgical techniques which were presented by their respective authors in a scientific manner as described. Francisco *et al*. reported many data in relation to their surgical technique of manual phacofragmentation using specially designed nucleotome in 50 eyes of 50 patients.[3] Preoperative and postoperative endothelial counts were evaluated, incidence of intra and postoperative complications (like intraoperative intracameral bleeding, postoperative corneal edema, iritis, raised IOP) were enumerated, postoperative astigmatisms were determined. Take another example. Akura *et al*., who used claw vectis for nucleus delivery, presented in their article[4] the results of their technique on 620 eyes of 510 patients. Therefore any new maneuver/ instrumentation in the anterior chamber should be backed by adequate data regarding its safety and efficacy before being advocated for large-scale public application.
